# Chronic Lymphocytic Leukemia as an Unusual Cause of Rapid Airway Compromise

**DOI:** 10.1155/2017/8247353

**Published:** 2017-03-15

**Authors:** Adrian R. Bersabe, Joshua T. Romain, Erin E. Ezzell, John S. Renshaw

**Affiliations:** ^1^Department of Medicine, Hematology/Oncology, San Antonio Military Medical Center, Fort Sam Houston, TX, USA; ^2^Department of Medicine, San Antonio Military Medical Center, Fort Sam Houston, TX, USA; ^3^Department of Medicine, Hematology/Oncology, Eglin Hospital, Eglin AFB, FL, USA

## Abstract

Chronic Lymphocytic Leukemia (CLL) is the most prevalent form of non-Hodgkin's lymphoma (NHL) in Western countries predominantly affecting adults over the age of 65. CLL is commonly indolent in nature but can present locally and aggressively at extranodal sites. Although CLL may commonly present with cervical lymphadenopathy, manifestation in nonlymphoid regions of the head and neck is not well described. CLL causing upper airway obstruction is even more uncommon. We describe a case of a patient with known history of CLL and stable lymphocytosis that developed an enlarging lymphoid base of tongue (BOT) mass resulting in rapid airway compromise.

## 1. Introduction

Chronic Lymphocytic Leukemia (CLL) is the most common form of adult non-Hodgkin's lymphoma (NHL). This mature B-cell neoplasm has an incidence rate of 4.6 per 100,000 people in the United States each year [1–5]. This disease process predominantly affects adults over the age of 65 and is more common in men. CLL has an extremely variable course of presentation ranging from asymptomatic lymphocytosis to painless lymphadenopathy, hepatomegaly, splenomegaly, cytopenias, and infections. Patients may also present with the typical “B” symptoms of unintentional weight loss, fever, and drenching night sweats [3–5]. Rapid progression with transformation into an aggressive, high-grade NHL is known as Richter syndrome and may occur in up to 10% of patients with CLL [6].

She was started on chemotherapy with bendamustine and rituximab (BR) with a dramatic anatomic response after two cycles (Figure 2) and near complete response (CR) after four cycles when tracheostomy was removed. She eventually completed 6 cycles of BR in October 2012 and achieved a CR with complete marrow recovery confirmed by bone marrow biopsy. She has been closely followed in the outpatient setting with no evidence of disease recurrence as of March 2017.

Although indolent in nature, CLL can present as locally aggressive extranodal mass resulting in symptoms depending on the location and extent of tissue involvement [7]. We present a unique case of a patient with known CLL and stable lymphocytosis for many years that later developed rapid airway compromise from an enlarging lymphoid base of tongue (BOT) mass which was identified as nontransformed CLL.

## 2. Case Presentation

A 62-year-old woman with untreated Rai stage II CLL was initially diagnosed in January 2007. At that time she was found to have an absolute lymphocyte count (ALC) of 8 g/dL, mild splenomegaly, and mild abdominal lymphadenopathy. She was otherwise asymptomatic. Bone marrow biopsy confirmed a monoclonal B-cell population of lymphocytes with CD5 and dim CD23 coexpression. Results at that time also showed normal cytogenetics, FISH negative for 11q deletion, 13q deletion, p53 deletion, and trisomy 12. *β*-2 microglobin was 2.4 mg/L and ZAP 70 was normal. She underwent close observation for several years. During this period of surveillance, she did not experience any recurrent bacterial infections, and serial monitoring of quantitative immunoglobulins demonstrated IgG levels greater than 500 mg/dL.

However, in December 2011, she presented to the emergency department for complaints of feeling a globus sensation over the span of forty-eight hours. This symptom was associated with dysphagia, diffuse myalgia, high-grade fevers, and shortness of breath. ALC at the time remained stable at 8.5 g/dL with no evidence of anemia or thrombocytopenia. Lactate dehydrogenase (LDH) was normal at 188 IU/L and *β*-2 microglobin was elevated to 3.3 mg/L. CT scan of the neck demonstrated a large heterogeneously enhancing 4.1 cm mass involving the palatine tonsils causing severe narrowing of the hypopharynx. She was admitted and quickly developed worsening shortness of breath and stridor prompting otolaryngology consultation for urgent tracheostomy placement.

The BOT mass was biopsied at the time of tracheostomy placement. The pathology specimen demonstrated squamous mucosa with acute inflammation and reactive hyperplasia most consistent with bacterial infection and lymphoid tissue hyperreactivity. No monoclonal lymphocyte population was identified. Furthermore, no pathogens were isolated from biopsy specimen, blood cultures, or sputum cultures, and CRP was normal at 0.06 mg/dL. However, the decision was made to empirically treat her with ten days of moxifloxacin. After completing her course of antibiotics, she noted complete resolution of her globus sensation, dysphagia, fevers, and myalgia.

**Figure 1:**
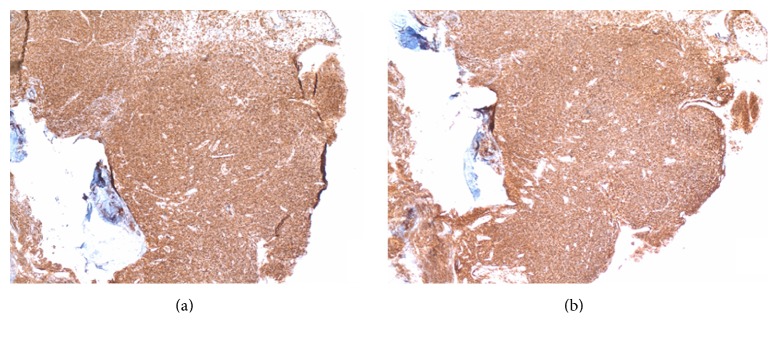
Immunohistochemical staining demonstrating a monomorphic population of CD20 positive lymphocytes with coexpression of CD5 typical of CLL.

**Figure 2:**
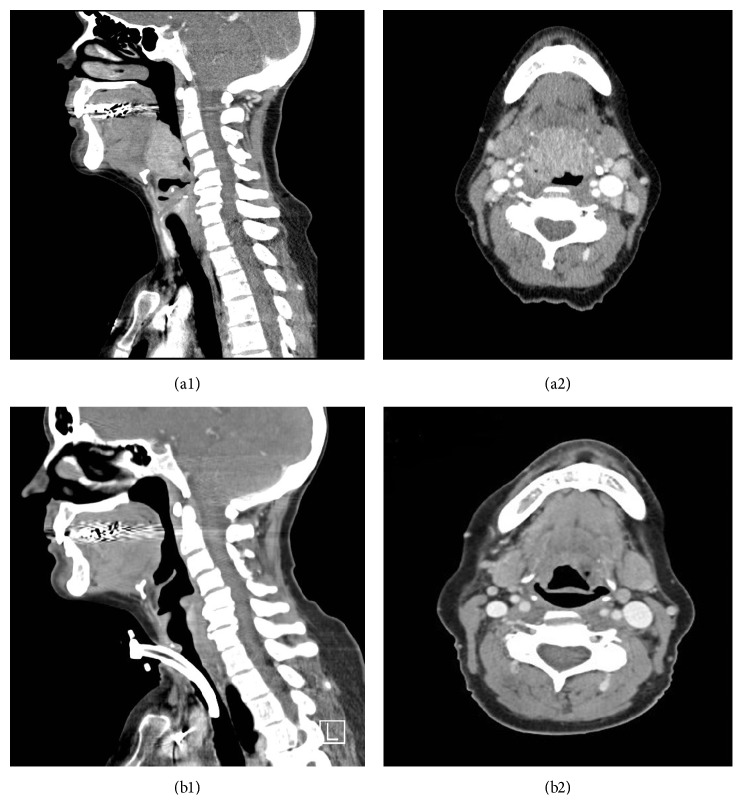
Interval change in size of BOT mass on contrasted CT scan of the head and neck before (a1 and a2) and after (b1 and b2) two cycles of bendamustine and rituximab.

Although her symptoms had completely resolved, a CT of the neck was repeated and showed persistence of the BOT mass six weeks after her initial admission for rapid airway compromise. Direct laryngoscopy was thus performed, and repeat biopsy of the mass now showed a monomorphic population of CD20+, CD5+, CD23+, and cyclin D1 negative lymphocytes consistent with patient's previous diagnosis of CLL (Figure 1). PET-CT to evaluate other sites of disease showed FDG-avid lingular tonsillar irregularity and hypertrophy (SUV of 4.9, mediastinal blood pool activity with SUV of 1.5) with numerous subcentimeter cervical lymph nodes that were only mildly FDG-avid (maximum SUV of 2.2). There was no other evidence of lymphadenopathy, and the spleen was not significantly enlarged. Given the patient's initial presentation with rapid deterioration resulting in respiratory failure, Richter transformation was considered. Again, however, biopsy results did reveal transformation into a more aggressive NHL clone, and PET-CT showed localized disease both of which argue against this phenomenon, however.

## 3. Discussion

Current literature regarding the presentation of CLL of the head and neck is very limited [5]. Although lymphomas often present in Waldeyer's ring of the pharynx, involvement of the oral cavity, larynx, and hypopharynx is exceedingly uncommon [7]. A rare case of palatal infiltration has been described by Vibhute et al. When lymphomas present in the oral cavity, some more common manifestations include gingival hypertrophy, petechial hemorrhage or ecchymosis, infection, ulceration, and necrosis [8]. Most presentations of CLL of the head and neck manifest as cervical lymphadenopathy or skin involvement, with the latter predicting poor prognosis [5, 8].

Upper airway obstruction resulting from CLL is even more uncommon. Although rare, airway obstruction is more often the result of mass effect from hilar and mediastinal adenopathy [4]. More common malignant causes of upper airway obstruction include locally advanced lung cancer as well as metastases from thyroid, skin, head and neck, or breast cancer [7, 9]. Regardless of the exact etiology, the approach to malignant airway obstruction is variable across different clinicians and institutions and may involve single or multimodality therapies [9].

The possibility of Richter syndrome with transformation of CLL into a large cell lymphoma was indeed entertained after it was later discovered that the underlying etiology of the patient's respiratory decline was due to CLL. Richter transformation can occur in 3 to 10% of patients with CLL. Underlying infections have also been implicated in triggering these transformations [6]. Although it is still debated, it is theorized that the Epstein-Barr virus (EBV) has a role in Richter transformation through direct oncogenic stimulation or through mitogenic and activating signals within the microenvironment. Rapid clinical deterioration is often a hallmark of Richter transformation similar to the presentation of our patient. The usual presentation of this aggressive transformation often includes increasing lymphocytosis, progressive cytopenias, elevated LDH, and sometimes diffuse, bulky lymphadenopathy [6, 10]. Other than rapid respiratory decline, our patient did not have any of these findings, and a repeat biopsy result confirmed CLL.

Another clinical challenge faced in this particular case was the presentation of a likely overlying infection given acuity of onset, fevers, and initial biopsy denoting acute inflammation and reactive lymphoid hyperplasia with a lack of monoclonal B-cell population. Infections are indeed the leading cause of death in patients with CLL and can account for up to 60% of disease-related mortality. This is due to a constellation of immune defects involving T-cell dysfunction, reduced complement levels, hypogammaglobulinemia, and impaired B-cell function [10]. A superimposed infection could not be ruled out in this case, given the initial presentation with acute inflammatory changes and lymphoid hyperplasia on biopsy and clinical improvement with antibiotics. In fact, Salem et al. describe four cases of patient with CLL who presented with B-symptoms and laboratory and imaging findings concerning progression of their lymphoma but were instead found to have a superimposed Herpes simplex viral infection. Our patient presenting symptoms concerning an underlying infection, but biopsy and microbiology work-up did not reveal a definitive pathogen.

CLL and other indolent extranodal lymphomas (ENL) are highly responsive to systemic therapy. For this reason, systemic therapy is considered the primary treatment modality for aggressive, localized ENL, unless they are considered unfit for or refuse such treatment. The treatment regimen of bendamustine and rituximab was chosen in this case due to high response rates and favorable safety profile [3]. However, low-dose radiation therapy is highly effective and may be considered for patients with stage IIIE/IV disease for palliative intent [7].

In summary, this case illustrates an unusual presentation of CLL causing airway compromise in a patient with a nontransformed indolent lymphoma. Prompt recognition is required to institute early intervention. Initial biopsy findings were concerning a possible bacterial infection in this patient with known CLL. However, a superimposed bacterial infection may have complicated the clinical picture. Nevertheless, cases of infection mimicking CLL progression have been described and accurate identification of the underlying etiology is essential in order to make appropriate treatment decisions. This patient responded very well and achieved a CR after completing six cycles of BR. She has maintained a CR for over four years after completing cytotoxic therapy.
